# Book Reviews

**Published:** 1914-11

**Authors:** 


### BOOK REVIEWS

Books mentioned may be inspected at and ordered through this office. So far as possible, books received in any month will be reviewed in the issue of the second month following.

**Local Anaesthesia, Its Scientific Basis and Practical Use.** Prof. Dr. Heinrich Braun, Zwicka; translated and edited by Percy Shields, M. D., A. C. S., Cincinnati. Lea & Febiger, Philadelphia and New York. 399 pages, 215 illustrations, including colored plates. $4.25

One of the most practical points about this book is the discussion of the scientific basis of local anaesthesia. The thorough understanding of the physiology of pain and the anatomic details connected with it afford a general conception of the problem of local anaesthesia which is scarcely dispensable in the successful choice and employment of practical methods. The special seat of painful sensations in the skin during operations, the indication to block afferent nerve channels in preventing pain, the relation of pain to shock, the intrinsic dangers of general anaesthetics, the total absence of mortality and injury to tissues in local anaesthesia, are important items discussed. As may be inferred merely from the size of the work, not only are different methods of local anaesthesia fully discussed but details necessary to the successful use of these methods in operations upon particular organs and regions are systematically presented. It is worthy of note that Dr. Baum himself is the artist who has prepared the illustrations for the work. Unfortunately few authors have the requisite talent for illustrating and the difficulty of impressing upon an artist the ideas to be conveyed, is too well known to need reiteration.

**A Manual of Practical Hygiene.** For students, physicians and Health officers. By Charles Harrington, M. D., late Professor of Hygiene in the Medical School of Harvard University. Fifth edition, revised and enlarged by Mark W.

Richardson, M. D., Secretary to the State Board of Health of Massachusetts, in collaboration with the following officials connected with the Massachusetts State Board of Health: W. H. Clark, Chief Chemist; X. H. Goodnough, Chief Engineer; William C. Hanson, M. D., Assistant to the Secretary; Hermann C. Lythgoe, Chief Analyst of Food and Drug Department, and George H. Martin, formerly Secretary to the Massachusetts State Board of Education. Octavo, 933 pages, with 125 engravings and 24 plates in colors and monochrome. Cloth, $5.00, net. Lea & Febiger, Publishers, Philadelphia and New York, 1914.

This is one of the most comprehensive works that has been presented on this subject. The first 224 pages are devoted to foods, although rather from the toxicologic standpoint and with special reference to their influence on health, than from the nutritional value appropriate to works on dietetics. The further discussion of alkaloid-containing, alcoholic and other beverages, condiments, spices and bakers' chemicals, food preservatives and contamination of food by metals, prolongs this section to 284 pages. Air, soil and water follow, up to page 483. The disposal of sewage and garbage, heating, lighting, ventilation, etc., disinfection bring the work up to page 631. Personal, occupational, school, military, naval and marine, tropical hygiene, follow. The control of infections, vital statistics and disposal of the dead make up the balance of the book. It will thus be seen that very little has escaped the author's attention. In spite of the complexity of subjects discussed and the opportunities for difference of opinion, the author has shown good judgment and thorough familiarity, throughout the work.

---

**Progress Report of the International Joint Commission** in re the pollution of Boundary Waters; including report of the Sanitary Experts. Jan. 16, 1914.

The Commission reports that at least five waterways must be investigated as involved in the mutual relations of Canada and the U. S.: Niagara River, Detroit River, etc. between Lake Huron and Lake Erie, St. Mary's River, St. Lawrence from Lake Ontario to a point where it departs from the boundary, and part of the St. John's River. Elaborate tables of bacteriologic investigation and charts showing average pollution at various points are given. For instance, for the Niagara River, the averages in colon bacilli per 100 c. c. (Phelps's method) were 0.5 in Lake Erie above local sources of pollution, 35600 opposite Strawberry Island on the American side, 1.6 on the Canadian side; 7500 in the American channel below the Tonawandas, 2000 below the Falls, 5714 at Lewiston and 14000 at the mouth. The pollution of Niagara is held to extend 10 to 16 miles into Lake Ontario. The old idea that water purifies itself by flowing—or rather, perhaps, by subsidence, is admirably demonstrated in a quantitative sense although the persistence of small but dangerous quantities of bacteria in general and of colon bacilli in particular demonstrates how little practical dependence should be placed on this factor. For example, in the Niagara River below the International Bridge at Niagara Falls the counts range from 10,000 near shore on either side, to 100 in the middle while, a few hundred feet further down stream, they range from 1000 to 100. Curiously enough, opposite Buffalo the count increases and decreases rather inconsistently with reference to the sewer outlets, apparently on account of the entry of highly concentrated sewage in the waters of the creeks which, in the aggregate, do not contain as much actual faecal matter as the trunk sewers. This reminds us of the point which we have previously made that the colon bacillus may indicate comparatively harmless contamination from animal sources, in other cases. The obvious lessons regarding water purification are presented, in reference to the Tonawandas, Niagara Falls and various other places. The Commission is to be complimented for the energy with which it has begun the task set before it. Such work is not only of direct benefit but, since it represents the mutual rights of two different countries, it establishes authoritative precedents, to compel sanitary reform generally.

---

**A Text-Book of Pathology.** For Students of Medicine. By J. George Adami, M. A., M. D., LL. D., F. R. S., Professor of Pathology in McGill University, Montreal, and John McCrae, M. D., M. R. C. P., (London), Lecturer in Pathology and Clinical Medicine in McGill University, formerly Professor of Pathology in the University of Vermont. Second edition, enlarged and thoroughly revised. Octavo, 878 pages, with 395 engravings and 13 colored plates. Cloth, $5.00, net. Lea & Febiger, Publishers, Philadelphia and New York, 1914.

It affords us special pleasure to review a book whose senior author is a member of our editorial staff. The work is divided, nearly equally into general and special pathology. Aside from the excellence of the illustrations, the use of diagrams to elucidate Mendel's law, immunity—in which case the diagrams give a good idea of the cogging of different factors—fertilization, etc., deserves special mention. The sub-contents, equivalent to tabula views under the chapter headings facilitate both reference and logical appreciation of relations. The chapter on the cell and the classification of infections neoplasms and monstrosities are particularly good. Partly on account of the growth of actual knowledge but partly, too, on account of clear literary style and didactic skill, a good many subjects formerly generally considered difficult and complicated by students, appear easy and simple. Ease and simplicity of the finished product always represent hard work on the part of the one who has prepared it. The profession owes a debt to the authors for the admirable manner in which they have performed their task.

---

**A Manual of Diseases of the Nose and Throat.** By Cornelius G. Coakley, M. D., Clinical Professor of Laryngology in the College of Physicians and Surgeons, Columbia University, New York. New (5th) edition. 12mo, 615 pages, with 139 engravings and 7 colored plates. Cloth, $2.75, net. Lea & Febiger, Publishers, Philadelphia and New York, 1914.

The author speaks, almost apologetically of having selected from the great mass of therapeutic measures advised, only those which he considers the best. The work is quite strictly limited to the nose and throat except that considerable attention is devoted to the various antra, several of the colored plates illustrating transillumination. Lesions due to syphilis, the exanthemata, etc., are carefully described. A general review of therapeutic measures concludes the book. The work is systematic and thorough without pretending to supplant some of the more detailed and extensive treatises.

---

**Manual of Diseases of the Eye,** for students and general practitioners. Dr. Charles H. May, New York, eighth edition, 440 pages, 377 original illustrations, including 22 plates and 71 colored figures. $2.00 Wm. Wood & Co., N. Y.

The modest implication that the book is not adapted to the use of the specialist depends rather on its comparative brevity than on other factors. It is sufficiently extensive and comprehensive for the use of any but a specialist requiring the most elaborate work of reference. It enters into bacteriology, pathology and the relations of ophthalmology to general disease and, at the same time, discusses practical problems in diagnosis and treatment.

---

**Progressive Medicine,** Vol. 16, No. 3, September 1, 1914. Edited by H. A. Hare, M. D., and L. F. Appleman, M. D., of Philadelphia, published by Lea & Febiger, Philadelphia .and New York, quarterly, $6.00 per annum. 339 pages.

The present number contains a review of progress in Diseases of the Thorax by Wm. Ewart; Dermatology and Syphilis by Wm. S. Gottheil; Obstetrics by Edward F. Davis; Nervous System by Wm. G. Spiller. While based on a review of current literature, the matter is presented in systematic form and thoroughly digested. The great value of this quarterly scarcely needs reiteration.

---

**Diseases of the Skin, Including the Acute Eruptive Fevers.** By Frank Crozer Knowles, M. D., Instructor in Dermatology in the University of Pennsylvania; Clinical Professor of Dermatology, Women's Medical College of Pennsylvania; Fellow of the College of Physicians of Philadelphia, etc., Octavo, 546 pages, with 199 engravings and 14 plates. Cloth, $4.00, net. Lea & Febiger, Publishers, Philadelphia and New York, 1914.

The inclusion of eruptive fevers in this work enhauces its value to the general practitioner, though of somewhat doubtful propriety, from the standpoint of the specialist and his relations to the general practitioner. On the other hand, the discussion of syphilis which is sometimes protracted to an undue degree if one limits skin manifestations of this disease to the dermatologic specialty, is comparatively brief. The work is well written and eminently practical. The metric prescriptions are rather too literal translations of the apothecary's formulae.

---

**Practical Therapeutics.** With Especial Reference to the Application of Remedial Measures to Disease and their employment upon a rational basis. Mr. Hobart Amory Hare, M. D., B. Sc., Professor of Therapeutics, Materia Medica and Diagnosis in the Jefferson Medical College of Philadelphia. New (5th) edition, thoroughly revised and rewritten. Octavo, 998, pages with 144 engravings and 7 plates. Cloth, $4.00 net. Lea & Febiger, Publishers, Philadelphia and New York, 1914.

The number of the edition attests the deserved popularity of this work, which ranks with Gray's Anatomy in the extent of its use. While the revision has been thorough, and new matter has been added especially in the discussion of vaccine therapy, the author has wisely followed the general plan of previous editions. The author is both scientific and practical and conservative both in adhering to drugs of established merit and in avoiding too great enthusiasm over new methods.

---

**Practical Medicine Series,** comprising ten volumes on the year's progress in medicine and surgery, under the general editorial charge of Charles L. Mix, A. M., M. D., and Roger T. Vaughan, Ph. B., M. D.; published by the Year Book Publishers, 327 S. LaSalle Street, Chicago. Price for the series of 10 volumes, $10.

Vol. 4. Gynaecology, edited by Emilius C. Dudley, A. M., M. D., and Herbert M. Stowe, M. D., 232 pages, $1.35 separately.

Vol. 5. Paediatrics edited by Isaac A. Abt, M. D.; and Orthopaedic Surgery, edited by John Ridlon, A. M., M. D., and Charles A. Parker, M. D., 228 pages, $1.35 separately.

Vol. 6. General Medicine, edited by Frank Billings, M. S., M. D., and J. H. Salisbury, A. M., M. D., 358 pages, $1.50 separately.

These volumes of the 1914 series maintain the standards of excellence set by previous issues and are commended to the profession.

---

**Reference Hand Book of the Medical Science, Third Edition,** edited by Thomas Lathrop Stedman, A. M., M. D., of New York. Published by Wm. Wood & Co., N. Y. Complete in 8 volumes. Vol. 4 (Emb-Hay). Price per volume $7 cloth; $8 leather; $9 half morocco. This volume contains 919 pages, 977 half-tone and wood engravings and numerous chromilithographs.

In spite of the quarter of a century which has elapsed since the issue of the first edition, we still find it of great value, not only in regard to anatomy and historic points, but for reference along many other lines. The new edition, upon the frame work of the two previous ones, ably edited and prepared by a large corps of collaborators, to bring each item up to date forms a cyclopaedia of medicine as complete as is, humanly speaking, possible. Our territory is represented in the present volume by Dr. F. Parke Lewis and the editor.

